# Can cell salvage be used for resuscitation in a patient with amniotic fluid embolism and hepatic laceration? A case report

**DOI:** 10.1186/s12884-022-04572-8

**Published:** 2022-03-26

**Authors:** Ping Li, Linli Luo, Dong Luo, Rurong Wang

**Affiliations:** 1 Department of Anesthesiology, West China Second University Hospital, Sichuan University, Chengdu, 610041 Sichuan China; 2 Key Laboratory of Birth Defects and Related Diseases of Women and Children (Sichuan University), Ministry of Education, 610041 Chengdu, P. R. China; 3 Department of Anesthesiology, West China Hospital of Sichuan University, No. 37, Guoxue Road, Sichuan 610041 Chengdu, P. R. China

**Keywords:** Amniotic fluid embolism, Intraoperative cell salvage, Hepatic laceration, CPR, Case report

## Abstract

**Background:**

Amniotic fluid embolism (AFE) is a rare disease that can lead to profound coagulopathy and hemorrhage, especially when combined with the laceration and bleeding of other organs. Intraoperative cell salvage (ICS) has been widely used for treating obstetric hemorrhage, but it remains unclear whether ICS can be used in the treatment of AFE.

**Case presentation:**

We report the case of a 27-year-old woman at 39 weeks’ gestation who suddenly developed severe abdominal pain, convulsions, loss of consciousness, and decreased vital signs during labor. Despite an emergency cesarean section being performed, the parturient experienced sudden cardiac arrest. Fortunately, the heart rate spontaneously recovered after effective cardiopulmonary resuscitation (CPR). Further abdominal exploration revealed right hepatic laceration with active bleeding. ICS was performed and the salvaged blood was promptly transfused back to the patient. Subsequently, the patient was diagnosed with AFE based on hypotension, hypoxia, coagulopathy, and cardiac arrest. The patient was transfused with 2899 mL salvaged blood during surgery with no adverse effects. At 60- and 90-day follow-ups, no complaints of discomfort or abnormal laboratory test results were observed in the mother or the baby.

**Conclusion:**

ICS was used to rescue patient with AFE, and ICS did not worsen the condition of patients with AFE. For pregnant women who received CPR, clinicians should explore the presence of hepatic laceration which can be fatal to patients.

## Background

Amniotic fluid embolism (AFE) is a rare disease which has an incidence of approximately 1/40,000 [1]. Although AFE is a rare disease, it is a potentially fatal condition, as well as a leading cause of unpredictable maternal death in developed countries [2]. Typical clinical symptoms of AFE include hypoxia, hypotension, and coagulopathy [1]. Moreover, with rapid progression, it may cause cardiac arrest and death [1]. The coagulopathy observed in AFE may differ from that observed in other forms of obstetric hemorrhages. It presents as coagulation dysfunction without significant hemoglobin decrease, because it may primarily be a depletion rather than dilutional process [3]. However, as the disease progresses, the number of red blood cells also decreases. The diagnosis of AFE may be delayed by a lag in laboratory tests on coagulation function [4]. AFE diagnosis is a type of exclusive diagnosis. When a patient exhibits the above symptoms, the clinician should actively initiate supportive treatment [1]. Patients with AFE may recover within a few hours if immediate resuscitation measures are taken upon the onset of transient hemodynamic decompensation and coagulopathy. Maintaining hemodynamic support and blood infusion is important during AFE interventions and ICS focuses on returning ample red blood cells (RBCs) to the patient in time. ICS has been widely used in obstetrics [5]. However, previous research on ICS for rescuing patients with AFE is scarce [3]. Here we present the case of a woman with obstetric hemorrhage who was diagnosed with AFE and was treated with ICS.

## Case presentation

A 27-year-old woman (gravida 1, para 0) visited a local clinic for delivery at 39 weeks’ gestation. At admission, the patient’s heart rate was 70 beats/minute, blood pressure was 120/78 mmHg, respiratory rate was 18 breaths/minute, and oxygen saturation was 98% in room air. Initial laboratory analysis demonstrated unremarkable results for platelet count, hemoglobin level, routine coagulation, and liver and renal function tests. Apart from gestational diabetes, fatty liver, and polyhydramnios, the patient had no known previous specific disease. She had regular uterine contractions after inducing labor with dinoprostone suppositories. However, she suddenly complained of abdominal pain and difficulty in breathing, followed by convulsions and loss of consciousness. Eclampsia was suspected and she was intravenously administered with magnesium sulfate. Ventilation with a mask was difficult as the oxygen saturation had reduced. The patient’s heart rate, blood pressure, and oxygen saturation gradually decreased. Accordingly, because of fatal heart rate decrease and no improvement in the patient’s vital signs, an emergency cesarean section was performed under general anesthesia and blood samples were collected for laboratory tests. Before delivery, the surgeon found no hemoperitoneum, but the nurse noted continuous bleeding from venipunctures. Ephedrine administration with tracheal intubation resulted in gradual improvement in vital signs of the patient. Anesthesia was maintained with oxygen and sevoflurane at approximately 1.5–2.5 vol%. Intraoperative mechanical ventilation was set to a volume control ventilation mode with a tidal volume of 450 mL, peak airway pressure of 25 mmHg, and respiratory rate of 16 beats/minute. A live fetus was delivered within 5 min. The 1-min and 5-min Apgar scores of the neonate were 4 and 6, respectively. The neonate was endotracheally intubated and transferred to the neonatal ICU.

**Fig. 1:**
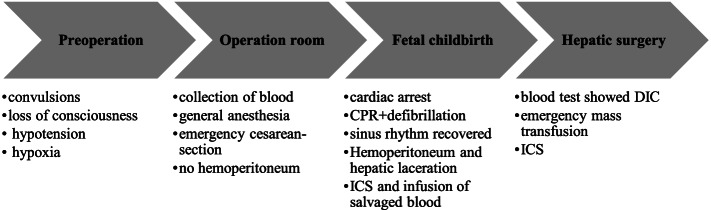
Intraoperative events. CPR: cardiopulmonary resuscitation. ICS: intraoperative cell salvage. DIC: disseminated intravascular coagulation

Immediately following delivery, the patient experienced cardiac arrest and cardiopulmonary resuscitation (CPR) was performed. After an epinephrine injection and defibrillation, the patient's sinus rhythm was recovered. After the uterus was closed, hepatic laceration in Section VII of the right liver with active bleeding was found during abdominal exploration. Vascular surgeons were immediately called for help for vascular and liver suture, and ICS (Haemonetics, cell saver, Elite,USA) was performed with two tubes for attracting blood at the same time. About 225 mL of salvaged blood was automatically washed with 1500 mL of NaCl (0.9%) (including 37.5 U/mL of heparin). The ratio of blood to heparin was about 1:7. The washing speed was 500 mL/min, and the salvaged blood was infused back through leukocyte depletion filters (Kangfu, China), which were replaced approximately every 600 mL to ensure the infusion speed and safety. Part of the salvaged blood was promptly transfused back to the patient. During hepatic surgery, surgeons observed bleeding without clotting and blood test results showed a serious decline in coagulation function with no decrease in hemoglobin levels (blood was collected at the beginning of the operation; Table 1, Fig. 1). Loss of consciousness, hypotension, hypoxia, cardiac arrest, and serious coagulopathy led to the diagnosis of AFE. Accordingly, emergency mass transfusion was initiated to correct the coagulation disorders and bleeding. Furthermore, calcium gluconate, bicarbonate, hydrocortisone, granisetron, ephedrine, phenylephrine, omeprazole, insulin, and furosemide were intraoperatively administered to the patient. The hemodynamics of the patient were then stable. The operation lasted for 347 min; blood loss was approximately 14,075 mL, including 5450 mL of autologous blood that underwent ICS. A large proportion of blood products were transfused (as shown in Table 1), including 30 units of RBCs, 10 g fibrinogen concentrate, 3250 mL fresh frozen plasma, 800 units of prothrombin complex, 5 units of platelets, 31 units of cryoprecipitate, and 2899 mL of blood was transfused back to the patient after ICS. The bleeding stopped after the coagulopathy was corrected and the liver laceration was repaired. After surgery, the patient was transferred to the ICU. At 60- and 90-day follow-ups, there were no complaints of discomfort or abnormal laboratory test results for the mother or the baby.

**Table 1:** Laboratory results and interventions around the time of operation

	HGB, g/L	Platelet, 10^9^/L	PT, s	aPTT, s	Fg, mg/dL	Intervention
On admission	130	232	10.4	27.6	379	
0.5 h post	122	119	> 150	> 300	< 50	6 units of RBC 4 g fibrinogen 800 units of Prothrombin complex 1 g calcium gluconate 250 mL (5%) NaHCO3 1 g tranexamic acid
1 h post	63	109	> 150	> 300	< 50	15 units of RBC 1900 mL FFP 17 units of cryoprecipitate 4 g calcium gluconate 2 u/h insulin
2 h post	73	36	16.1	82	131	9 units of RBC 950 mL FFP 4 g fibrinogen 14 units of cryoprecipitate 4 units of platelets 2 g calcium gluconate 250 mL (5%) NaHCO3
3 h post	96	86	14.5	57.3	184	400 mL FFP 2 g fibrinogen 1 unit of platelets 3 g calcium gluconate 10 mg furosemide
5 h post	124	103	15.6	44.4	211	ICU

0.5 h post: 0.5 h after AFE

*HGB* hemoglobin, *PT* prothrombin time, *aPTT* activated partial thromboplastin time, *Fg* fibrinogen, *RBC* red blood cells, *FFP* fresh frozen plasma

## Discussion and conclusions

AFE is a potentially fatal disease and the main cause of unpredictable maternal death. There is no gold standard diagnostic method for AFE, and to date, an exclusive diagnosis approach is followed for AFE. The diagnosis depends on bedside evaluation and judgment. When patients experience sudden loss of consciousness and convulsions, clinicians are likely to first consider eclampsia or brain disease. However, cardiac arrest and profound deterioration of coagulation function without a decrease in hemoglobin levels led us to suspect AFE over eclampsia. Coagulation test results often lag, so clinical coagulopathy should be considered [4]. Fortunately, in this case, cardiac arrest occurred after delivery and effective CPR recovered circulation in the patient, which increased the success rate of rescue for both the mother and the fetus. However, liver lacerations were observed following CPR, which may be linked to increased liver congestion, fatty liver, coagulation dysfunction, and physiologic compression of the liver capsule during pregnancy [6]. A previous study indicated that the incidence of liver lacerations following CPR was higher in pregnant patients than in the general population [6]. Accordingly, early consideration for diagnosis may allow for prompt treatment of this life-threatening complication. Unfortunately, both coagulation disorders following AFE and hepatic laceration increased the risk of acute hemorrhaging. Therefore, supplementation with RBCs and coagulating substances was urgently needed. ICS can supply abundant RBCs in time but has not been widely used in patients with AFE.

ICS technology can reduce the need for allogeneic blood transfusion. Several reports have shown that routine use of ICS in obstetric procedures does not cause “iatrogenic” AFE [5, 7]. AFE occurs when the maternal–fetal barrier is destroyed during delivery, resulting in exposure of the mother to fetal tissues, which subsequently causes clinical symptoms. Multiple studies have indicated that trophoblastic tissue, the protein elements, α-fetoprotein, and tissue factor, which is a clotting promoter believed to contribute to coagulation dysfunction and seen in increased concentrations in patients with AFE, can be effectively removed by modern cell salvage equipment and processing [7, 8]. However, there is no research report on whether these risk factors remain in the salvaged blood of patients with AFE after washing. Thus, it is recommended to test the composition of autologous blood before and after washing.

We transfused salvaged blood to treat massive bleeding without knowing that AFE had occurred. Blood was recovered with almost complete absence of amniotic fluid after delivery, separate leukocyte depletion filters were used to ensure appropriate infusion speed and safety, and a total of 2899 mL autologous blood was transfused. No adverse reactions occurred during infusion. So far, in only one study a patient with AFE was reported who was transfused with salvaged blood, but that patient developed hypotension after salvaged blood infusion, which improved after stopping the infusion [3]. Moreover, several studies reported that leukocyte depletion filters might contribute to significant hypotension during transfusion in obstetric surgery, and the hypotension was not specific to patients with AFE alone [9, 10].

ICS was used to rescue patient with AFE, and ICS did not aggravate the patient’s condition or cause adverse reactions. Thus, in pregnant women who have received CPR, the clinician should identify the presence of hepatic laceration, which can be fatal to patients.

## Data Availability

The datasets obtained and/or analyzed during the current study are available from the corresponding author on reasonable request.

## Abbreviations

AFE: Amniotic fluid embolism
RBCs: Red blood cells
ICS: Intraoperative cell salvage
CPR: Cardiopulmonary resuscitation
HGB: Hemoglobin
PT: Prothrombin time
aPTT: Activated partial thromboplastin time
Fg: Fibrinogen
FFP: Fresh frozen plasma

## References

[1] Pacheco LD, Saade G, Hankins GD, Clark SL, Society for Maternal-Fetal Medicine (SMFM), Electronic address: pubs@smfm.org (2016). Amniotic fluid embolism: diagnosis and management. Am J Obstet Gyneco.

[2] Fitzpatrick KE, Tuffnell D, Kurinczuk JJ, Knight M (2016). Incidence, risk factors, management and outcomes of amniotic-fluid embolism: a population-based cohort and nested case-control study. BJOG.

[3] Rogers WK, Wernimont SA, Kumar GC, Bennett E, Chestnut DH (2013). Acute hypotension associated with intraoperative cell salvage using a leukocyte depletion filter during management of obstetric hemorrhage due to amniotic fluid embolism. Anesth Analg.

[4] Ponzio-Klijanienko A, Vincent-Rohfritsch A, Girault A, Le Ray C, Goffinet F, Bonnet MP (2020). Evaluation of the 4 diagnosis criteria proposed by the SMFM and the AFE foundation for amniotic fluid embolism in a monocentric population. J Gynecol Obstet Hum Reprod.

[5] Goucher H, Wong CA, Patel SK, Toledo P (2015). Cell Salvage in Obstetrics. Anesth Analg.

[6] Cox TR, Crimmins SD, Shannon AM, Atkins KL (2018). Liver lacerations as a complication of CPR during pregnancy. Resuscitation.

[7] Hayata E, Nakata M, Takano M (2021). Biochemical effects of intraoperative cell salvage and autotransfusion during cesarean section: A prospective pilot study. J Obstet Gynaecol Res.

[8] Bernstein HH, Rosenblatt MA, Gettes M, Lockwood C (1997). The ability of the Haemonetics 4 Cell Saver System to remove tissue factor from blood contaminated with amniotic fluid. Anesth Analg.

[9] Waldron S (2011). Hypotension associated with leucocyte depletion filters following cell salvage in obstetrics. Anaesthesia.

[10] Kessack LK, Hawkins N (2010). Severe hypotension related to cell salvaged blood transfusion in obstetrics. Anaesthesia.

